# The promise of equal education not kept: Specific learning disabilities – The invisible disability

**DOI:** 10.4102/ajod.v9i0.647

**Published:** 2020-02-26

**Authors:** Melanie A. Gow, Yvonne Mostert, Lorna Dreyer

**Affiliations:** 1 Department of Educational Psychology, Stellenbosch University, Cape Town, South Africa

**Keywords:** specific learning disabilities, higher education, inclusive education, PRISMA, transformation, equality

## Abstract

**Background:**

This research is part of a larger project on the exploration of inequalities in South African higher education. This current study focussed on the implementation of policies to eradicate inequalities in an inclusive education system.

**Objectives:**

This article aimed to establish the implementation of policy by researching the lived experiences of students with specific learning disabilities (SLDs) studying in the university.

**Method:**

A qualitative, systematic review was employed as the research methodology. Original peer-reviewed qualitative studies published between 1994 and 2017 were systematically reviewed. The Preferred Reporting Items for Systematic Reviews and Meta-Analyses (PRISMA) was used to ensure rigorous reviews. The Critical Appraisal Skills Programme (CASP) was used to guide the process of critical appraisal of the selected articles which resulted in a total of 10 articles being selected for reviewing. The target population of this research comprised undergraduate students diagnosed with SLD. Semi-structured interviews were the main data collection tools used in the studies that were reviewed. Data from the selected articles were extracted and synthesised.

**Results:**

The dominant themes that emerged from the review were: (1) fear of stigmatisation; (2) gaps in policy implementation; (3) experiences vary across departments; and (4) self-determination and family support as success factors.

**Conclusion:**

An important aspect in the transformation of higher education institution is to ensure the closing of the disjuncture between policy and implementation in support of students with SLD.

## Introduction

The creation of a democratic society in which social structures promote unity in diversity in pursuit of transformation in South Africa is inextricably linked to the debate on inclusive education. Various operational guidelines for the transformation of universities and the promotion of social inclusion have been developed since 1994. Against the backdrop of a demised Apartheid-framed education system, it is not surprising that the focus of transformation in higher education is on increasing access to and participation of black students as compared to former whites-only institutions. The drive and efforts to transform universities in South Africa are thus mainly focussed on increasing racial equality through incentives such as the National Student Financial Aid Scheme (NSFAS) to support students who come from disadvantaged and impoverished backgrounds (Department of Higher Education and Training [DHET] 1999). The rationale for this funding mechanism is to increase access to higher education and meet ‘equity, growth and quality targets’, as aspired to in the White Paper on Higher Education and Training (DHET 2013:8) and the National Development Plan (The Presidency, 2011:325).

Within the South African context, the Department of Education (DOE) uses the broad concept of ‘barriers’ to learning within a single inclusive education system (DOE 2001:6). Barriers to learning refer to a range of factors, including disability, which impede on access to and participation in educational institutions (DOE 2001). However, although there is a rich body of research on experiences of students with disabilities in higher education, it is not afforded the same level of significance as diversifying the racial profile of access to universities in South Africa. Conversely, universities do have policies to support students with disabilities who enter higher education. The focus, though, is on physical accessibility of buildings for students with physical disabilities, while special attention, such as extra writing time during tests and examinations, is also awarded to accommodate and support students with special needs (Dreyer 2011).

As a subproject on the exploration of inequalities in South African higher education, this qualitative systematic review (QSR) focussed on the lived experiences of students with specific learning disabilities (SLDs), with a focus on access and participation in the university. The review included literature published from 1994 to 2017. The research question that guided this research was: what are the transition experiences of undergraduate students with SLDs when moving from high school to higher education?

### Transforming higher education

The education ministry (DOE 1997) envisioned a transformed and democratic higher education system, which will:

[*P*]romote equity of access and fair chances of success to all who are seeking to realise their potential through higher education, while eradicating all forms of unfair discrimination and advancing redress for past inequalities. (section 1.14)

Not surprisingly, the first point on the transformation agenda in Education White Paper 3 (DOE 1997) is to increase and broaden participation of all marginalised groups. Central to the DOE’s argument, therefore, is the emphasis on successful policy implementation to overcome the legacy of fragmentation, discrimination and inadequacy (section 1.13). The DOE’s vision is to:

[*I*]ncrease access for black [people], women, disabled and mature students, and generate new curricula and flexible models of learning and teaching, including modes of delivery, to accommodate a larger and more diverse student population. (DOE, 1997: section 1.13)

Aligned with the vision to increase and broaden educational access and participation, Education White Paper 6 (DOE 2001) presents a framework for establishing such an inclusive education system with the emphasis on special needs education. White Paper 6 embraces inclusive education as part of the broader social reform of the South African education system. It builds on the Bill of Rights in which the democratic values of human dignity and equality regardless of race, ethnicity, gender, disability and religion are enshrined. The National Development Plan (2011) and higher education policies such as White Paper 3 (DOE 1997) make further attempts to realise this transformation agenda. With a focus on equity, quality and access, South Africa embraces the notion of education for all (EFA) (UNESCO 2007) in its overall social, political and economic transformation initiatives. Concurring with this is Pantić and Florian’s (2015) assertion that inclusive education is concerned with improving the quality of mainstream education and reducing the disparities in its achievement outcomes.

At an international level, there is also an increasingly strong drive to widen access to higher education institutions (HEIs). This drive is backed by a strong political agenda to decrease social marginalisation and social inequality with ‘appropriate legislation that is designed to ensure non-prejudiced practice within higher education institutions’ (Kendall 2016:3).

Sound policies and guiding documents for implementation are, however, only a starting point to the transformation process and may provide the impetus for change to which the society reacts. According to Ebo (2016), the move towards inclusive education brought about an increase in students with disabilities enrolling in universities internationally. Higher education institutions needed to embrace and accept students with disabilities in the academic space, and the need to make buildings more accessible to, for example, wheelchairs and the other more visible disabilities was highlighted. However, SLDs result in less visible barriers to learning and has motivated universities to develop policies for the support of students with special learning needs.

### Specific learning disabilities

The Diagnostic and Statistical Manual of Mental Disorders, Fifth Edition (DSM-5) (American Psychiatric Association [APA] 2013) provides definitions for and classifies mental disorders for use in diagnosis and treatment. According to DSM-5, SLD is a neurodevelopmental disorder that is ‘diagnosed when there are specific deficits in an individual’s ability to perceive or process information efficiently and accurately’ (APA 2013:32). Specific learning disability therefore affects one or more of the basic cognitive processes required for understanding or use of spoken or written language. The disorder may manifest itself in an inadequate ability to ‘listen, think, speak, read, write, and spell or to do mathematical calculations’ (APA 2013:32). Specific learning disability therefore impedes someone’s ability to learn or use specific academic skills that form the foundation for other academic learning. Such difficulties can have a long-term impact on a person’s ability to function in everyday life, particularly in academia that involves activities and mastery with numbers, written words and written expression (APA 2013).

It must be noted that the APA uses the medical term ‘specific learning disorder’ for diagnosis. The term ‘learning disability’ is, however, commonly used in educational and legal systems. Although learning disability is not exactly synonymous with a specific learning disorder, someone with a diagnosis of specific learning disorder can expect to meet the criteria for a learning disability and have the legal status of a person with a recognised disability to qualify for accommodations and services in academic institutions (APA n.d.). According to the Learning Disability Association of America (LDA) (n.d.), people who present with learning disabilities are generally of average or above average intelligence. However, there seems to be a gap between the person’s potential and actual accomplishment. Because there are no visible indications of a disability, SLDs are often referred to as ‘hidden’ or ‘invisible’ disabilities. The LDA classifies the following conditions as SLD:

- Auditory-processing disorder
- Dyscalculia
- Dysgraphia
- Dyslexia
- Language-processing disorder
- Non-verbal learning disabilities
- Visual perceptual or visual motor deficit.

Other related disorders include attention deficit hyperactivity disorder, dyspraxia, and executive dysfunction and memory disorder. However, the LDA cautions not to confuse learning disabilities with learning problems. Learning problems primarily result from visual, hearing or motor handicaps; intellectual disability; emotional disturbance; or environmental, cultural or economic disadvantages.

### Barriers to participation

The United Nations Convention on the Rights of Persons with Disability (UNCRPD) Article 24 states that persons with disabilities should be guaranteed the right to inclusive education at all levels, regardless of age, without discrimination and on the basis of equal opportunity (United Nations [UN] 2008:16). As a neurodevelopmental disorder, SLD is caused by intrinsic factors associated with the medical discourse on disabilities (UN 2008). Although there is an international move away from a medical perspective of viewing disabilities, the authors contend that biological and thus medical factors can be responsible for creating barriers to learning and participation. However, these barriers may be exacerbated by society and institutional systems. Therefore, from a social model perspective of disabilities, it is posited that social, environmental and attitudinal barriers can be incapacitating (Kendall 2016). The social model furthermore states that society’s response to the presence of an impairment imposes a disability upon the person. It is thus imperative that educational institutions, more specifically universities, find ways to negate the negative impact of such societal barriers on students with SLD.

Promoting inclusion of students with SLD at universities is a complex issue. Research has indicated that social inclusion (formal access to university) does not automatically equate to epistemological access for students with SLD (Dreyer 2017; Morrow 2007). There are several barriers that limit access to the knowledge imparted in lecture halls at the university. These barriers can be systemic as well as pedagogical. At a systemic level, barriers are increasingly being addressed through policy development. Practical implementation of policy can overcome systemic barriers by driving programme adjustments to support students in meeting academic requirements (Mercer & Mercer 1998). A focus on pedagogy, referring to the methodologies and practices used in teaching, implies that adjustments within the teaching space can help overcome barriers to epistemological access. These adjustments generally include (1) extended time to complete the programme, (2) course substitution, (3) modification or waiver of a foreign language requirement, (4) part-time study and (5) extended time for tests. However, such adjustments tend to be generic and put most of the onus on the individuals who have to declare their disability to the university. As SLDs are invisible, such declarations are required to be supported by psychological assessments (Couzensa et al. 2015:25).

With the move towards inclusive education and inclusive teaching practices (pedagogy that allow for epistemological access), the focus is less on how to get the individual to fit into the system but rather on employing inclusive pedagogy to teach all. While much has been written about inclusive pedagogical practices at the school level, there is limited literature on the topic in relation to higher education. Morrow (2007) used the concept of ‘epistemological access’ (p. 18) to refer to the accessibility of various forms of knowledge imparted at learning institutions. Institutional barriers, for example, negative attitudes or a disabling educational environment, may impede access to learning in a higher education educational setting for students with an SLD (Ryan 2007). Recently, researchers have shown that universities are developing policies to establish support services. However, while student support services within universities are generally regarded positively, the support plans are considered useful but ‘generic’ and do not consider individual differences (Kendall 2016).

At the school level in South Africa, as in many countries worldwide, inclusive education is supported by policies for identification and support (Burr et al. 2015; Department of Basic Education 2014). South Africa has recently adopted the screening, identification, assessment and support (SIAS) strategy to be implemented in all schools from 2017 (Department of Basic Education 2014). The strategy enables early identification of barriers to learning in order to provide appropriate support to learners. Currently, universities in South Africa do not have a similar policy to continue the recognised provision of support.

The research question that guided this QSR was therefore: what are the transitioning experiences of undergraduate students with SLD moving to higher education? The question was formulated with the specific aim to review literature on the experiences of undergraduate students with SLDs as they transitioned to higher education.

## Methodology

According to Vergnes et al. (2010), a qualitative ‘systematic review of the literature is the scientific way of synthesising a plethora of information, by exhaustively searching out and objectively analysing the studies dealing with a given issue’ (p. 771). There has been an increase in QSRs in the field of education (Andrews 2004). According to Bearman et al. (2012:625), QSRs provide a clear, well-organised and thorough approach to a literature review and the synthesis of the research findings. A QSR is a research methodology used to review, evaluate and synthesise existing research to answer a research question. For this QSR, the guidelines presented by the Preferred Reporting Items for Systematic Reviews and Meta-Analyses (PRISMA) were used to ensure rigorous reviews (Moher et al. 2009b). The 27 items proposed by PRISMA are subdivided into the title (1), abstract (2), introduction (3 and 4), methods (5–16), results (17–23), discussion (24–26) and funding (27). Each item is accompanied by a description as a checklist item. In addition, the PRISMA flow diagram (Moher et al. 2009a) was used to guide this review process.

This QSR reviewed original, peer-reviewed, qualitative studies published from 1994 to 2017 that focussed on the experiences of undergraduate students with SLD. This period was selected as 1994 marked a significant year in South African history, namely, the dawn of a democratic political dispensation with an emphasis on transformation of educational institutions and a specific focus on access for all. Research articles from this period were included to investigate inclusive practices and how students experienced it over the last two decades.

### Search strategy

The literature search was conducted during July and August 2017. The following keywords were used in the search of databases listed for educational psychology and curriculum studies on the Stellenbosch University Library Guides site: SLD; higher education; undergraduate experiences; transition to university; accommodations for SLD; and physical access and curriculum access. Only English language articles were included in the search. The search was conducted on four different databases. The initial search yielded 974 possible articles. Additional filters (time frame: 1994–2017; peer-reviewed and qualitative research) were added to narrow the search, and the databases yielded the following results:

ERIC: 71 articles

ProQuest: 78 articles

Google Scholar: 23 articles

SAGE Journals: five articles

After the additional filters were applied, the search yielded a total number of 177 articles.

In the second phase of the search, the articles were screened by title, and duplicates were excluded (*n* = 55). The next phase was focussed on screening abstracts for eligibility to be included. Screening criteria included the target groups, time frames, type of study and text selection (4T’s) to be consistent with the research question and focus of this study. A final number of 20 studies were selected to be included in the review.

### Methodological quality appraisal

During the fourth phase of selection, the full texts of the 20 selected studies were assessed for methodological quality with the use of the Critical Appraisal Skills Programme (CASP UK n.d.). The full-text articles were reviewed and assessed by two independent reviewers to determine whether the article adequately met the criteria for inclusion in the QSR. The 10 questions in the CASP qualitative check list are designed to help researchers systematically read the full texts while considering three broad questions:

1. Are the results of the study valid?
  - Was there a clear statement of the aims of the research?
  - Is a qualitative methodology appropriate?
  - Was the research design appropriate to address the aims of the research?
  - Was the recruitment strategy appropriate to the aims of the research?
  - Were the data collected in a way that addressed the research issue?
  - Has the relationship between the researcher and participants been adequately considered?
2. What are the results?
  - Have ethical issues been taken into consideration?
  - Was the data analysis sufficiently rigorous?
  - Is there a clear statement of findings?
3. Will the results help locally?
  - How valuable is the research?

**FIGURE 1:**
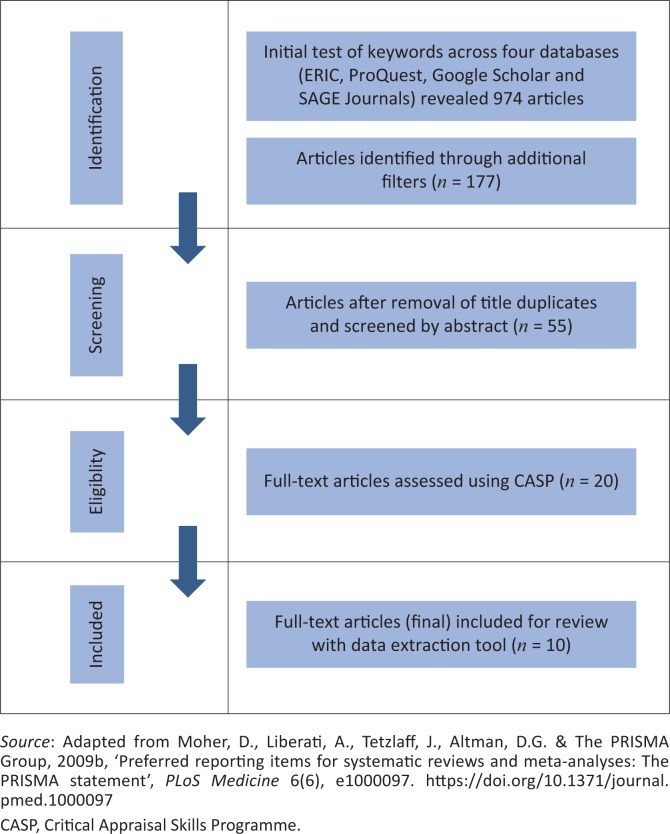
Preferred reporting items for systematic reviews and meta-analyses flow diagramme.

These questions supported the rigour of a critical appraisal of the selected articles. If all the answers to the questions for an article were YES, the article scored 100%. During the methodological quality appraisal, articles with a score of 0–79 were excluded and those with a score of 80–100 were included. A total of 10 articles were selected to be reviewed. The scores obtained for each of the included articles are indicated in the data extraction table (Table 1). The process followed in this QSR is presented in Figure 1.

**TABLE 1:** Data extraction table.

Descriptors	Title	Data collection methods	Participants	Country	Aim	Findings	CASP score
Anctil, Ishikawa and Scott (2008)	Academic identity development through self-determination (successful college students with learning disabilities)	Semi-structured interviews	Nineteen undergraduate students with learning disabilities	The United States of America	To explore the cognitive and behavioural manifestations of self-determination in successful college students with learning disabilities	Persistence and a powerful desire to succeed contributed to success This was enhanced by acceptance of one’s disability, understanding one’s strengths or weaknesses and goal achievement Family support contributed to positive academic identity	100
Strnadová, Hájková and Květoňová (2015)	Voices of university students with disabilities: Inclusive education on the tertiary level – a reality or a distant dream?	Semi-structured interview: lived experience	Sample of 24 students with learning disabilities	Czech Republic	Student experiences regarding barriers to access to education at the tertiary level, to ascertain existing support and explorestrategies to overcome barriers	Barriers created by lack of understanding and lack of inclusive practice approaches Barriers experienced include institutional attitudinal barriers and disability-specific barriers Some lecturers refuse lectures to be audio-taped. Note-taking is difficult while following lectures or notes, and PowerPoint slides are unavailable before class Support mainly from family, peers and assistants Found that students use the following strategies to overcome the barriers at university: assertiveness; self-determination; metacognition; efforts to fit in; optimism and career planning	90
Ryan (2007)	Learning disabilities in Australian universities: Hidden, ignored and unwelcome	Semi-structured interview: lived experience	Eight students responded voluntarily to an invitation to participate	Australia	Highlights how higher education’s non-inclusive practice can affect the experiences of students with learning disabilities	Policies are in place, but ‘Despite requests for assistance, such as the provision of lecture notes, adjustments to assessment tasks, or merely asking their lecturer to go at a slower pace, more often than not these were not provided’ (p. 438) Lack of understanding by academic staff Grades not reflecting their ability They reported difficulties in dealing with the ‘copious amounts of reading materials’ (p. 440), the pace of lectures and the inability to ask questions, reduced personal contact with lecturers and fewer opportunities for clarification, and the reliance on large amounts of reading and writing for most assessment tasks Feelings of being less supported than in high school Constantly having to ‘explain and provide evidence’ (p. 439) of difficulties experienced Strong feelings of a lack of understanding, of acceptance and even of legitimacy	80
Couzensa et al. (2015)	Support for students with hidden disabilities in universities: A case study	Semi-structured interviews	Small-scale study of students with learning disabilities	Australia	To investigate the experiences of students with learning disabilities regarding access to universities	Informal networks and family were stated as most effective. Some experienced caring and flexible lecturers and tutors Mixed reactions to the value of universal design approaches to support Difficulty in accessing resources for support Assistive technology difficult to use Barriers for providing the discipline-specific supports	90
Luna (2002)	Learning from diverse learners: (Re) writing academic literacies and learning disabilities in college	Interview and focus group discussion sessions: lived experience	Small-scale study of students with learning disabilities	North Eastern United States	To identify experiences of students with learning disabilities in order to better understand and to improve the support needed to increase access	Teaching and learning practices experienced as standardised and formal Teaching practices in universities do not embrace diversity and inclusivity. If success is not achieved, then the problem lies with the student Student–lecturer relationships further disempower those students who require support by restricting their access, and therefore their participation in meeting their own learning needs The students did not feel heard or accommodated	80
Kendall (2016)	Higher education and disability: Exploring student experiences	Semi-structured interview: lived experience	Sample of 13 students with learning disabilities	The United Kingdom	To explore student experiences with learning disabilities with regard to inclusive practice	A reluctance to disclose a disability because of perceived associated stigma Student support services within the university were viewed as a positive resource ‘Learning support plans were considered useful but too generalised in terms of particular disabilities’. (p. 9) Barriers were identified as: staff being unaware of a student’s disability, unwillingness to make reasonable adjustments and a lack of assessment choice	90
Stage and Milne (1996)	Invisible scholars: Students with learning disabilities	Semi-structured interview: Lived experience	Small-scale study of eight students with learning disabilities	Midwest United States	Explored experiences of college students with learning disabilities and the ways in which they were accommodated and how they adjusted to college	Revealed character traits as well as institutional factors that affected their experiences Negative self-image and perception had to be overcome and the development of strategies to cope were necessary Although experiencing success, it was evident that the universities needed to be more inclusive in their overall knowledge and understanding of learning disabilities and the teaching and learning implications Points to self-determination as a factor for success	90
Holloway (2001)	The experience of higher education from the perspective of students with disabilities	Semi-structured interviews	Sample of six students with learning disabilities	The United Kingdom	Investigation into the experiences of students with learning disabilities at the university	The findings revealed that students generally experience a model that fails to recognise disability holistically, and is still individualised. In spite of an inclusive policy, students still experience marginalisation and disempowerment. Dyslexia is recognised individually but nothing officially Informal support by administrative staff is valuable while it is difficult to set a meeting with academic staff. While extra time is allowed for examinations, students found that for internal examinations it is decided on somewhat arbitrarily Support from academic staff was experienced ‘from highly supportive and aware, to cynical, unhelpful and non-consultative in decision making which directly affected them’ (p. 605) Where ‘staff lacked awareness or specific knowledge of the disability and support available’ (p. 605), adverse feelings were reported	90
Gibson (2012)	Narrative accounts of university education: Sociocultural perspectives of students with disabilities	Two semi-structured interviews conducted 6 months apart	Sample of five students with learning disabilities	Southwest of England	To relay the lived experiences of students with learning disabilities in relation to effective learning practices and inclusion in the transition from high school to higher education	This research suggests that university practitioners are not as well-prepared (inclusive) as they could be in meeting particular needs Institutional structures, values and practices impact on inclusion of students with learning disability mostly led to frustration Friendships and peer group play an important role in academic success; as well as some teaching approaches that involve small groups	90

*Source:* Critical Appraisal Skills Programme UK (CASP UK), n.d., *CASP checklists*, viewed 30 August 2018, from https://casp-uk.net/casp-tools-checklists

CASP, Critical Appraisal Skills Programme.

### Ethical considerations

Ethical clearance has been received for the larger project. This qualitative literature review does not require ethical clearance. However, it adhered to ethical considerations stated in the article (ethical clearance number: EFEC 1-6/2017). This article reports on a QSR and thus constitutes meta-research. However, Vergnes et al. (2010) cautioned against ignoring ethical considerations or at least to avoid the risks associated with systematic reviews such as including studies that do not respect ethical principles. Ethical considerations for this meta-research meant that all the studies included reported voluntary participation. They also report on receiving informed consent from participants, and pseudonyms were used to protect the identities of participants. However, it must be noted that some of the original articles included may have had ethical transgressions as not all reported explicitly that they received institutional permission to conduct the research. Permission was only implied by stating ethical considerations observed.

### Data extraction

Comprehensive data extraction (Higgins & Green 2011) was conducted by identifying and describing five general descriptors in each of the selected articles. The descriptors were title, data collection, target group, country of research, aims of the study and the findings. The descriptors of each article are presented in Table 1.

At this stage, it is important to note that although the literature search yielded literature on access of students with disabilities from South African studies, none focussed on the experiences of students with learning disabilities and were thus not included in this report.

### Data synthesis

The findings from the articles reviewed were summarised and presented in a narrative form. Qualitative content analysis (Creswell 2014) was used to inform data synthesis. In conducting the synthesis, the authors were cognisant of the inclusion criteria and the research question that guided this study. Using an inductive approach, patterns in the data were identified by means of thematic codes (Creswell 2014; Patton 2002). We identified the themes recurring throughout the reviewed literature. These were then described and listed.

The dominant themes that emerged with regard to experiences of students with SLD during transition from high school into higher education included:

- fear of stigmatisation
- gaps in policy implementation
- variation of experiences across departments in the same HEIs
- self-determination and family support as success factors.

## Results

### Description of the studies

After the screening and methodological quality appraisal process, only 10 articles were included in the final review. All selected articles underwent the same processes of filtration, screening and appraisal with regard to the relevance of their titles and abstracts to the research question. The qualifying articles were authored in the following countries: the United States of America, the United Kingdom, Australia and the Czech Republic. The majority of studies (*n* = 4) were conducted in the United States of America.

Semi-structured interviews were the main data collection method used across studies. The participants in all studies were undergraduate students who had some form of SLD. All articles reported on small-scale studies that focussed in depth on the lived experiences of students with learning disabilities and their transition from high school to higher education. The main focus of articles was on access and inclusion into HEIs.

The dominant themes that emerged are listed under data synthesis above, and are discussed further.

#### Theme 1: Fear of stigmatisation

The experience of being misunderstood by lecturers and the feelings of guilt for requesting support, resulted in many students with SLD not disclosing their disability and their need for support. Negative self-perceptions, along with the reluctance of lecturers to recognise and support students with SLD through inclusive practices, were dominant experiences of students, and students generally experienced stigmatisation because of the disability. Consequently, many students did not feel comfortable revealing their learning disability to the lecturers.

#### Theme 2: Gaps in policy implementation

The reviewed studies indicated that the HEIs have inclusion policies in place. However, the general experience amongst students was that HEIs have not embraced (or yet understood) inclusion and are not meeting the diverse needs of their students. As a consequence, most students reported experiences of insufficient support or lack of support in the transition from high school to higher education. Many students also felt that the lecturers were not informed or equipped to support their needs, resulting in the students not feeling included. Strnadová et al. (2015:1092) concluded that lecturers and higher education staff should be trained further on supporting diversity.

According to the reviewed literature, students with SLD experience several barriers to academic access and participation. Lectures and administrative staff are, for example, not well-prepared to accept students with SLD. Consequently, students experienced that their particular needs were not met and that they did not have equal access to the curriculum, which limited their participation in learning. The following verbatim report by Jackie is representative of some students’ experiences at university (Ryan 2007):

I thought uni was going to be so good, [but] it was just a horror semester. I hated it. I did well, I got good marks, but it had nothing to do with the university, it was a statement about my application. (p. 439)

Most studies in this review revealed that the standardised teaching practices in universities do not acknowledge diversity or promote inclusivity and as such restrict academic access and participation. Students feel disempowered within the lecturer–student relationship and experience that they do not have a voice and are not being accommodated. In Kendall’s (2016) research, Grace voiced her experience as follows:

Sometimes when you ask for an extension, they are a bit begrudging and ask you, do you really need one? I wouldn’t be asking if I didn’t! It’s so annoying. (p. 8)

It further seemed that most lecturers attributed the difficulties that students experienced as arising solely from within the individual (a medical perspective) and did not see that their lecturing practices can have an impact as well.

Students felt that the policies and support provided for SLDs were tailored to serve the institution rather than the student. Furthermore, in their experience, barriers to access academia were exacerbated by a lack of resources within the institution.

#### Theme 3: Experiences vary across departments

Findings indicate that the experiences of students vary across departments in the same HEI, manifesting in different levels of inclusion. Some students experienced inflexibility and lack of resources for ensuring access. Kendall (2016) quoted a student’s experience when she asked the lecturer to provide lecture material before the class to help her make notes in class:

He said that he couldn’t do that because he had done that in the past and when people got the reading material, they didn’t turn up for the lecture! I should really have made a stand about it but I didn’t have the energy to argue. (p. 6)

Some students reported experiencing negative attitudes from lecturers towards them. While everybody did not experience negativity, the students reported inconsistent experiences across departments and with individual lectures.

#### Theme 4: Self-determination and family support as success factors

Self-determination has been associated with many positive results in students with SLD. Students acknowledged that their own tenacity and determination as well as their own knowledge regarding their disability and needs had been key factors in their success. Students were generally of the opinion that that their biggest support sources were family and friends. They felt that the lecturers misunderstood them, or made them feel guilty for asking for support. Most of the students relied on their peer group and family for support and did not seek the support that the higher institutions offered, as this support was experienced as inflexible.

## Discussion

Globally, there is a strong focus on inclusion as a means of widening access to education to overcome a history of fragmentation, inequality and inadequacy. The culmination of social and academic events on inclusion led to widespread acceptance of policies to ensure equal access to quality education. Researchers argue that there is ample evidence to support a statement that students with SLDs are enrolling in universities at an increasing rate (Couzensa et al. 2015; Ebo 2016; Kendall 2016). The promotion of inclusive practices in educational settings is increasingly gaining momentum and is supported and encouraged through several initiatives from the UN.

Early identification of and support for learning barriers are given priority in basic education in South Africa through the implementation of the SIAS strategy. Unfortunately, although there is an established body of literature on physical and sensory disabilities in South African HEI’s, none could be found for SLD during the search process used in this article. However, from the findings of this study, it is clear that at an international level, HEIs struggle to implement policies that are geared towards increasing inclusion. Although, much success is reported regarding the support of students with physical and sensory disabilities, they tend to lag behind in terms of providing support for students with SLD.

While barriers were identified by teachers in the basic education system, university-level policy requires students to disclose their disability in order to gain access to the support provided. This review found that students with SLD tend to refrain from disclosing their disability for fear of being stigmatised. Participants in the studies indicated that they fear negative perceptions from lecturers. This finding is corroborated by literature in that SLDs are not visible in the same way that sensory and physical disabilities are, and that students then have to prove the need for support by submitting an assessment report from a psychologist (Couzensa et al. 2015:25). Their fear of stigmatisation also seems to be confirmed when students experience reluctance from lecturers to recognise and support SLD through inclusive practices in class.

While universities have adopted disability supportive policies, this study shows that students with SLD still do not enjoy the same support as their physical and sensory disabled counterparts. The themes that emanated from this research show that university students with SLD are largely on their own with regard to coping with the academic demands of tertiary education. This seems to be true even against the backdrop of students having received support at the school level (Ryan 2007). The deduction can be made that policy implementation in the form of academic support in schools far outweighs support for students with SLD at the university. The current gap between policy on pedagogical inclusive practice and implementation in the higher education system seems to result in failing students who have the right to equal access to quality education. The findings further show that students with SLD find it difficult to access sources of support.

The inconsistency in how departments at the same university support students with SLD confirms the existence of a gap between institutional policies and implementation. However, it might also be possible that there is no uniform interpretation of policy amongst lecturing and administrative staff. A grave concern is that findings from the literature show that lecturers at the university have not adapted their own pedagogy in pursuit of inclusive education. The assumption that some lecturers persistently exclude students who are diagnosed with SLD through traditional lecturing styles is based on student experiences reported here. This traditional ‘one-size-fits-all’ approach tends to create barriers to knowledge for students with SLD. A ‘one-size-fits-all’ approach is further exacerbated by the disparate power relationship between lecturers and students, which leaves students with SLD feeling disempowered and voiceless. The students’ situation of powerlessness is compounded by the finding that students feel that the policies are developed to favour the institution and not those who need it.

It is interesting to note that because of the reported lack of resources and inflexible pedagogical practices, students tend to rely heavily on family and friends for emotional and other support. According to the literature reviewed here, support provided at universities is experienced as inflexible and does not adequately serve their specific individual needs. Therefore, students with SLD rely on their peers for specific support; for example, as quoted in one of the reviewed studies (Couzensa et al. 2015):

I found in the past year that the most successful strategy is becoming part of a study group … we give each other deadlines … we set up a reading schedule…you know that everyone else in the group is expecting your … summary of that chapter … that keeps you moving. (p. 33)

Ultimately, students declare that it is their own determination to succeed in spite of the barriers they experience that contributes to their success. Their tenacity and determination as well as their own knowledge of their disability and needs had been a key factor in their success.

### The challenge to make specific learning disability visible

The themes that emerged from this study indicate that efforts to offer and ensure equal opportunities and access for all remain a fundamental concern in higher education today. Students currently enrolled at university experience insufficient support in relation to what the basic education system provides. The invisibility of SLD has been described as detrimental to students’ success in higher education.

Internationally, several policies, declarations and policy implementation guidelines transpired with the central theme of inclusion and participation as essential to human dignity and the enjoyment and exercising of human rights. Such policies have given impetus to move away from a medical/deficit view of disability to viewing education as a human rights and social justice issue (Kendall 2016).

However, SLD is not an overt and easily understood disability. According to university policy, students are required to undergo assessments and then disclose the disability in order to gain access to and enjoy the support provided by universities’ disability units. Universities thus need to be proactive on several levels in order to make SLD visible. The findings of this review indicate that universities do have policies on inclusion of students with disabilities, including students with SLD. However, SLDs tend to be given less prominence than visible disabilities. This results in the needs of students with SLDs remaining undetected or undisclosed and thus ‘ignored’. A paramount problem seems to be the lack of policy implementation. Implementation is compromised by interpretation of the policies when departments at the same institution have varying attitudes and support in place.

One way of making SLD visible is by promoting awareness amongst the lecturing staff and support services. This can be done through several awareness and training initiatives that are geared towards inclusive practices in the institution as a whole.

Teacher training programmes are increasingly preparing student teachers to be able to teach in inclusive schools and to be responsive, pedagogically and socially, to the needs of all (Burr et al. 2015). When students with SLD enter university, they expect the same acknowledgement of SLD they experienced in school, only to be disillusioned by the pedagogical inflexibility of the university lecturers. This inflexible ‘one-size-fits-all’ pedagogical approach that most university lecturers still practice needs to be challenged. This can be done through in-service training on how to be pedagogically responsive to the needs of all the students in the class. Training at faculty and departmental levels can contribute to practices of implementation that acknowledge SLD, consequently making it more visible.

### Conclusion

The international drive to promote inclusive quality EFA is currently showing results in the form of policy implementation, particularly at the level of basic education. By addressing the pedagogical needs of students with SLDs at the school level, HEIs experience an increase in students with SLD. This increase in the enrolment of students with SLD in universities necessitates continued support in order for them to be successful in their studies.

There is ample evidence that universities have been implementing policies to ensure safe and equal access to those who have physical and sensory disabilities. This QSR has indicated that students with SLD undergoing higher education experience the effects of a disjuncture between policy and practices. The inflexible pedagogical practices of lecturers seem to be a major contributor to this disjuncture. Upon reflection, it is interesting to note that the articles by Greenbaum, Graham and Schales (1995), and by Stage and Milne (1996), which were published in the era of awareness campaigns and policy-making, revealed similar student experiences across the themes. On the other hand, more recent articles reflected greater diversity of student experiences. The most obvious differences are found in the students’ recommendations. At the time when these articles were published, access to higher education for students with learning disabilities was less likely than that of their peers (Forlin & Chambers 2017:556). The universities were not attempting to provide access and support to students with disabilities. Research recommendations thus focussed on improving access to information for lecturers and staff in higher education. It is of interest to the authors that in recent articles, more students with learning disabilities are attending HEIs and information on learning disabilities and inclusion is more readily available, yet pedagogical practices within institutions have not adapted to this new reality.

In the pursuit of overcoming historically determined patterns of fragmentation, inequality and inefficiency, SLD needs to be acknowledged and students must be supported. It is concluded that universities need to make SLDs visible by ensuring sound implementation of policies at all levels of the institution. The emphasis should, however, be on providing support and training for lecturing staff to enable them to provide meaningful access to the knowledge and skills they wish to impart in lectures through pedagogically responsive practices.

It is concluded that transformation in higher education towards support and the provision of epistemological access to all students remain imperative. Inequalities need to be addressed through ensuring policy implementation within the teaching and learning spaces. Specific learning disabilities need to become visible and accepted as a disability in universities if they are to be inclusive centres of excellence for all.
